# Struma ovarii with follicular thyroid-type carcinoma and neuroendocrine component: case report

**DOI:** 10.1186/1477-7819-10-93

**Published:** 2012-05-21

**Authors:** Federico Selvaggi, Domenico Risio, Mathew Waku, Daniela Simo, Domenico Angelucci, Alberto D’Aulerio, Roberto Cotellese, Paolo Innocenti

**Affiliations:** 1 Unit of General and Laparoscopic Surgery, Biomedical Sciences Department, “G. d’Annunzio” University, Via dei Vestini 31, Chieti, 66100, Italy; 2 Unit of Pathology, “G. d’Annunzio” University, Chieti, Italy

**Keywords:** Struma ovarii, Follicular thyroid carcinoma, Hysterectomy, Bilateral salpingo-oophorectomy

## Abstract

Struma ovarii (SO) is a slow-growing ovarian neoplasm with thyroid tissue as its predominant component. It is an uncommon neoplasm, usually asymptomatic with an unknown risk of malignant transformation. Due to difficulties in assessing the rare biological nature and the discrepancies in the reported cases, a consensus on the appropriate treatment has not been definitively reached.

A 50-year-old female was subjected to upper gut endoscopy which showed a 30-mm mass located in the gastric antrum, suggestive of mesenchimal tumor. Incidentally, a pelvic CT scan also documented a solid mass in the right adnexa, with morphological characteristics of ovarian neoplasm. The patient underwent gastrectomy, total hysterectomy, bilateral salpingo-oophorectomy with lymph node dissection, and omentectomy. Histology documented the presence of gastric cavernous angioma, and, in the right adnexa, foci of follicular thyroid-type carcinoma arising in SO with a well-differentiated neuroendocrine component.

Here we report and discuss the clinical and morphological presentation of follicular thyroid-type carcinoma arising in SO. The neoplasm was discovered incidentally and had a favorable clinical outcome at 1-year follow-up.

## Background

Struma ovarii (SO) is a monodermal variant of ovarian teratoma which predominantly contains thyroid tissue [1-4]. The incidence is from 0.1% to 0.3% of all ovarian teratomas [5]. Patients are usually asymptomatic or predominantly presented with a pelvic mass in 45% of cases or abdominal pain [5]. Menstrual irregularities and clinical hyperthyroidism have been demonstrated in 9% and 5% of cases, respectively [5,6]. Diagnosis of SO is based on histopathological criteria and guidelines for primary thyroid gland disease [2,5]. Malignant SO consists of papillary thyroid carcinoma (PTC) cells, follicular variant of PTC or follicular thyroid carcinoma (FTC) cells, and mixed follicular/papillary carcinoma cells [7,8]. The most common type is PTC, followed by FTC and the recent form of highly differentiated follicular carcinoma (HDFCO), characterized by extraovarian dissemination of thyroid elements [2,4,9]. The differential diagnosis between benign and malignant neoplasm is still extremely difficult, in particular for SO with a follicular growth pattern and undefined capsule. The incidence of malignancy is also difficult to assess due to the rare nature of this case and the absence of standardized diagnostic criteria [5,9]. Malignant transformation has been reported to vary from 5% to 37% of cases, metastasis is seen in 23% of cases and it is mainly intra-abdominal [5].

Basing our research on the review of specific scientific literature, we examined this uncommon case of follicular thyroid-type carcinoma arising in SO, unsuspected in the preoperative evaluation.

## Case report

**Figure 1:**
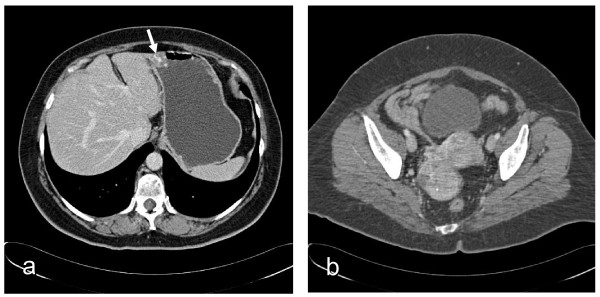
**CT appearance of gastric and ovarian neoplasms:** 30-mm solid mass with minute calcifications located between the anterior wall of corpus and gastric antrum (**a**, arrow). Pelvic mass of 65×45×60 mm on right adnexa suggestive of mixed cystic-solid ovarian tumor (**b**).

A 50-year-old female presented to our observation with epigastric pain. Her past surgical history was uneventful except for appendectomy. A 30-mm gastric solid mass, suggestive of mesenchimal tumor, was documented by endoscopy. Due to its high vascularization and the risk of gastrointestinal bleeding, a biopsy was not indicated. The CT scan of the abdomen confirmed the presence of the gastric lesion, and incidentally showed a pelvic mass of 65×45×60 mm in the right adnexa (Figure 1). The patient was asymptomatic. The pelvic finding was confirmed by US scan: the right ovarian mass had a prevalent solid component with a rich supply of blood vessels. No signs or symptoms of hyperthyroidism were observed. The patient underwent right oophorectomy, and after intraoperative histological diagnosis of ovarian carcinoma, a total abdominal hysterectomy with bilateral salpingo-oophorectomy, retroperitoneal lymph node dissection, and omentectomy were finally performed. The gastric lesion was resected and the digestive continuity was restored by Billroth’s II anastomosis. At surgery, no abnormal lymph nodes and ascites were noted. Histology did not reveal signs of peritoneal carcinomatosis in the pelvis. Macroscopically the right ovary mass measured 62x45 mm and cut sections showed a solid white-yellowish tissue with focally hemorrhagic areas.

**Figure 2:**
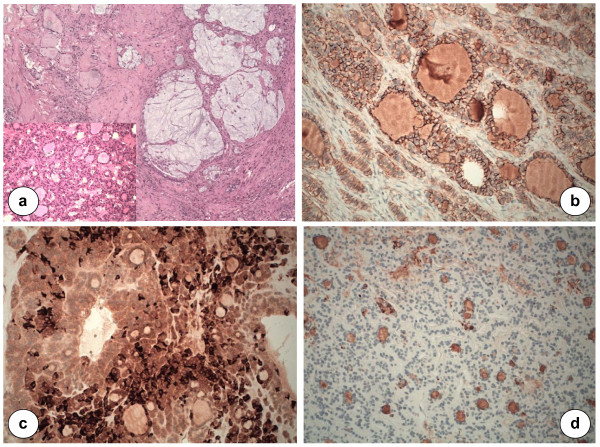
**Histological and immunohistochemical profile of thyroid type carcinoma in SO.** The sections show follicular thyroid cells with the mucoid glandular component (H&E), ×10 (**a**); strong and diffuse CD56 reactivity, ×20 (**b**); Chromogranin A expression in ovarian struma, showing the neuroendocrine cells × 20 (**c**); Intrafollicular material and the immunoreactivity for thyroglobulin, ×20 (**d**).

**Figure 3:**
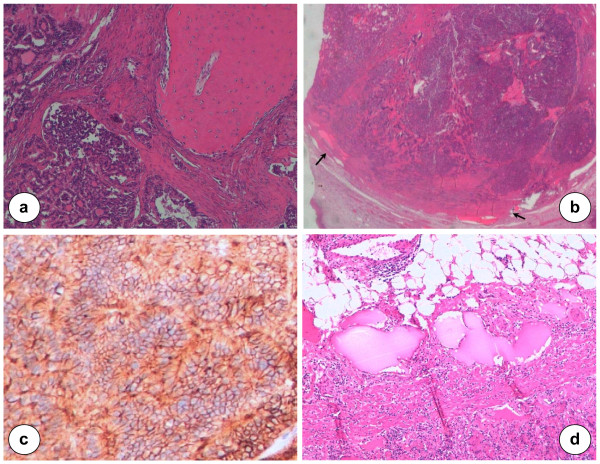
**Histological and immunohistochemical profile of thyroid type carcinoma in SO.** Microscopical relationship between SO and ovarian teratoma: on the right side mature bone tissue with osteoblasts; on the left, follicular thyroid cancer cells, ×20 (**a**). Vascular invasion: capillary structures are invaded by cancer cells in the pericapsular area (**b**, black arrows). Neuroendocrine tumor morphology with cordonal-alveolar pattern (**c**). Cavernous hemangioma of the stomach arising from submucosa: on the lower part of the section, normal gastric mucosa and muscolaris mucosae, ×20 (**d**).

The follicular thyroid-type carcinoma was characterized by the proliferation of cells arranged in follicular and trabecular pattern (Figure 2). In the contest of ovarian teratoma, the follicular component showed an infiltrative growth as observed in thyroid carcinoma with moderate differentiation. The mitotic index was not elevated and any necrotic tissue areas have been documented. The follicular tumor border was infiltrated but the cellular growth was restricted within the capsule. Vascular invasion was reported in pericapsular capillary structures (Figure 3). Together with the focal follicular thyroid-type carcinoma, the mature ovarian teratoma showed a well-differentiated neuroendocrine component with cells arranged in cordonal-alveolar structures (Figure 3).

For immunohistochemistry, sections of formalin-fixed paraffin-embedded samples were treated with H_2_O_2_/3% for 5 minutes to inhibit endogenous peroxidase and then washed in H_2_O. Antigen was unmasked by treatment with EDTA at pH 9, or with citrate buffer at pH 6 in a microwave oven (two 5-minutes courses). The slices were then held for 20 minutes at room temperature. After washing in PBS/Tween-20, sections were incubated for 30 minutes with the primary antibodies. Then, they were washed and stained with Bond^TM^ Polymer Refine/HRP Detection Kit according to the manufacturer’s protocol (Leica, Wetzlar, Germany) or Bond^TM^ Polymer Refine Red Detection Kit (Leica) for PgR, ER, TTF1, Chromogranin A, Calcitonin, CD56, CDX2, CEA, CK20. For negative controls, we substituted non-immune sera for the primary antibodies. The immunohistochemistry analysis demonstrated positivity for PgR, TTF1, Chromogranin A, CD56, CDX2, CEA, and CK20. It was negative for ER and calcitonin. Based on these specific immunophenotype profile, diagnosis of follicular thyroid-type carcinoma arising in SO was made (Figures 2 and 3). The multiple peritoneal biopsies and the lymph nodes were free from metastatic cancer cells. In addition, peritoneal washing revealed mesothelial cells, leukocytes but not tumor cells on cytological examination. The definitive diagnosis of gastric neoplasm was cavernous hemangioma involving submucosa (Figure 3).

The immediate postoperative course was characterized by anemia treated by medical supporting therapy and blood transfusions. Postoperative thyroid scan and thyroid function were normal. She was referred for thyroidectomy but she refused the operation. Thyroglobulin levels were monitored in the postoperative period. Nine months after treatment, laboratory evaluation revealed normal serum TSH (0.95 mU/L) and thyroglobulin (6.17 ng/mL) levels. Local and distant recurrences were not observed at the CT scan evaluation after a follow-up period of 1-year and the patients was scheduled for further follow-up. During this period the US of the neck revealed a normal thyroid gland.

## Discussion

Although SO has elicited considerable interest since it was first described, many diagnostic aspects are still unknown. SO, defined as containing 50% or more thyroid tissue, accounts for approximately 5% of all ovarian teratoma, with an incidence of malignant transformation reported in 5% to 37% of SO [1,10,11]. Most cases are found incidentally and for this reason the only clinical data are obtained from retrospective reports. Previous studies have demonstrated that the majority of patients with SO are asymptomatic or accompanied by non-specific symptoms similar to other ovarian neoplasms. At the time of diagnosis, the most common symptoms were lower abdominal pain, palpable abdominal mass, ascites, and abnormal vaginal bleeding [5,12]. Unusual clinical presentations such as hyperthyroidism and Meigs' syndrome have been also documented [2]. The incidence of hyperthyroidism was reported to be 5% to 8% and 17% to 33% of the cases had ascites at diagnosis [4-6,12,13]. The pathophysiology of hyperthyroidism in SO is still unknown. Matsuda and colleagues have reported that malignant SO can be diagnosed before operation by the evaluation of free T3, and T4, Thyroglobulin, and TSH. The extremely high levels of Thyroglobulin in local ovarian venous blood compared with that in peripheral blood provide evidence of the production of Thyroglobulin in the ovarian tumor and the normalization of it’s serum levels is related to surgical resection of the tumor [13]. In our case the patient did not show any specific symptoms and the diagnosis of SO was incidental and only established after surgical removal.

Thyroid tissue in SO is morphologically, biochemically identical to that of cervical thyroid gland. For this reason the diagnosis of malignant SO is in conformity to the same criteria used for thyroid carcinoma, such as the presence of ground glass nuclei, vascular invasion, and mitotic activity [3]. Unfortunately, the concept of malignant SO in the literature is confusing due to lack of standardized prognostic parameters. The diagnosis of a well-differentiated variant is particularly difficult due to lack of a well-defined tumor capsule in the ovarian tissue. In these cases, the presence of infiltration by tumor cells into the surrounding ovarian tissue, and involvement of vascular system, or metastasis, highly supported the diagnosis of malignancy [2]. Although the histological aspects of malignancy are often documented, the majority of patients that underwent surgical treatment for thyroid type carcinoma arising in SO did not show a clinically aggressive outcome with high rates of recurrences and metastatic spread. The biological behavior remains often enigmatic and seems not to be correlated with the observed long-term clinical outcomes.

With the efforts of understanding the prognostic parameters and defining more appropriate treatment care, a molecular and morphological consensus is advocated to standardize diagnostic criteria of malignancy in cases of thyroid type carcinoma in SO.

Distant metastasis has been reported to be a rare feature of SO in approximately 5% of cases [12]. Other clinical studies demonstrated a higher intra-abdominal metastatic rate of 23% [5]. Common metastatic sites are the omentum, peritoneum, lymph nodes, fallopian tubes, and the contralateral ovary [12]. Some patients presented with distant metastases to the lungs, bone, brain, liver, and mesenteric surfaces of the spleen and diaphragm [8,11]. In clinical diagnosis, thyroid type carcinoma in SO has to be differentiated from cystadenoma and other primary ovarian cancers or metastatic tumors. Generally, SO appeared as a smooth-margin multicystic mass with a high attenuation signals during pre-contrast on CT scan. Signal intensities on T1-weighted images were usually intermediate to high, and those on T2-weighted images were low as recently reported by Shen and colleagues [14]. The majority of tumors showed a mixed cystic and solid mass, with capsule wall thickness of 3 mm on average, and contained transparent, green, or brown fluids [14]. To our knowledge five lethal cases have been reported in literature with recurrences and metastatic disease [8]. In our case no metastasis have been documented during 1-year follow-up after surgery.

The treatment of malignant SO remains controversial and no consensus exists on the surgical and non-surgical modalities. Surgical treatment consists in total abdominal hysterectomy plus bilateral salpingo-oophorectomy with omentectomy and lymph nodes sampling, a reasonable therapy for postmenopausal women with diagnosis of carcinoma. In other conditions, especially when fertility has to be preserved, conservative surgery concerning unilateral oophorectomy might be proposed together with a strict follow-up [5]. This hypothesis should be carefully discussed with the patients. Laparoscopic surgery may offer some advantages in surgical staging and its mini-invasive removal but this technique is not standardized for these uncommon conditions [6]. After the initial surgery, some authors have advocated near total thyroidectomy and radioactive iodine ablation to detect and treat recurrent disease [11]. Total thyroidectomy is mandatory to exclude a primary thyroid neoplasm in the differential diagnosis of SO. Total thyroidectomy followed by ¹³¹ I radio ablation therapy should be reserved for patients with recurrence or residual disease [5,10]. Thyroglobulin is a well established marker for monitoring the recurrence of malignancy. An increased serum levels of Thyroglobulin represent the early detection of recurrence as reported in many studies [6,11].

SO containing thyroid type carcinoma must be distinguished from papillary or follicular thyroid carcinoma metastatic to the ovary [4]. It is mandatory to study the thyroid gland for the differential diagnosis of primary or secondary tumor of the ovary. De Simone and colleagues have proposed thyroidectomy to confirm normal thyroid gland, by excluding a primary thyroid carcinoma, and potentiate radioactive iodine therapy [3]. No consensus has been reached in performing prophylactic total thyroidectomy after the diagnosis of thyroid type carcinoma in SO. In this situation, the combination of surgical removal with subsequent thyroidectomy and radiotherapy do not represent a standardized therapy but it is a comprehensive therapeutic modality, still not supported by scientific data in reducing the risk of recurrence and increase prognostic indices of selected cases.

## Competing interests

The authors declare that they have no competing interests.

## Authors’ contributions

FS, MW, RC, and PI analyzed the data and wrote the manuscript. DR, AD, and DS participated in the acquisition and interpretation of radiological data. DA carried out the histological and bio-molecular studies. FS and PI contributed to the final version and carried out the clinical case report. All authors read and approved the final manuscript.
